# Fabrication of Chitosan-Based Intumescent Flame Retardant Coating for Improving Flame Retardancy of Polyacrylonitrile Fabric

**DOI:** 10.3390/molecules24203749

**Published:** 2019-10-17

**Authors:** Yuanlin Ren, Tian Tian, Lina Jiang, Yingbin Guo

**Affiliations:** 1 School of Textiles Science and Engineering, Tiangong University, Tianjin 300387, China; 1731015031@stu.tjpu.edu.cn (T.T.); jianglina1218@163.com (L.J.); gyb0936@163.com (Y.G.); 2 Key Laboratory of Advanced Textile Composite, Ministry of Education, Tiangong University, Tianjin 300387, China

**Keywords:** polyacrylonitrile fabric, photo-grafting polymerization, chitosan, glycidyl methacrylate, flame retardancy

## Abstract

In order to improve the flame retardancy of polyacrylonitrile (PAN) fabrics, glycidyl methacrylate (GMA) was first grafted onto the surface of PAN fabric (PAN-g-GMA) by means of UV-induced photo grafting polymerization process. Then, PAN-g-GMA was chemically grafted with chitosan to obtain a bigrafted PAN fabric (PAN-g-GMA-g-CS). Finally, the flame-retardant PAN fabric (FR-PAN) was prepared by phosphorylation. The structure and elemental analysis of the samples were characterized by Fourier transform infrared spectroscopy (FTIR) and X-ray photoelectron spectroscopy (XPS). The thermal degradation properties and combustion characteristics of the fabrics were accessed by thermogravimetric analysis (TG), differential scanning calorimetry (DSC), and cone calorimeter (CC). The results show that the onset thermal decomposition temperature of FR-PAN fabric is lower than that of the control sample due to the degradation of the grafting groups. The combustion test indicates that the FR-PAN fabric has an excellent flame-retardant property and the combustion rate is significantly reduced. In addition, the char residue of the burned FR-PAN fabric is over 97%, indicating excellent char-forming ability.

## 1. Introduction

Polyacrylonitrile (PAN) fibers, one of the most important synthetic fibers, have a good reputation of synthetic wool, and are widely used in the textile industry. Unfortunately, the limiting oxygen index (LOI) value of PAN fibers is ca. 17%, thus making it one of the most combustible synthetic fibers. Therefore, the development of flame-retardant PAN fibers or fabrics has become particularly important and attracted extensive attention of researchers.

Ultraviolet (UV) grafting technology has the advantages of low-cost, easy to control, mild reaction conditions, limited grafting to the surface of the material without destroying the matrix material, and has great industrial application prospects. It has been successfully applied to impart materials’ flame-retardant properties [1,2,3,4,5,6]. In our previous work, we used UV-induced photo-grafting technology to impart PAN fabric flame retardancy. For example, acrylic acid (AA) was UV-grafted onto PAN fabric followed by chemical modification to obtain fire-retardant PAN fabric [7]. The LOI value of the treated fabric after 20 cycles of washing was 27.3%, thus showing good flame-retardant durability. In addition, hydroxyethyl methacrylate (HEMA) was grafted onto the surface of PAN fabric, followed by phosphorylation to successfully obtain the flame-retardant PAN fabric with the LOI value of 32% [8]. Furthermore, glycidyl methacrylate (GMA) was UV-grafted onto the surface of PAN fabric, followed by ammoniation and phosphorylation [9]. The total heat release (THR) of flame-retardant PAN fabric decreased from 7.3 MJ/m^2^ to 4.6 MJ/m^2^, and the peak of heat release rate (PHRR) of the treated fabric fell from 374 kW/m^2^ to 162 kW/m^2^, indicating good flame-retardant performance. Similarly, the team prepared GMA-grafted PAN fabric, and the fabric reacted with hydrazine hydrate followed by phosphorylation, which exhibited good flame retardancy [10]. The THR and PHRR of the modified fabric declined by 38.4% and 60.2%, respectively. Moreover, the LOI value of the flame-retardant PAN fabric after 30 washing cycles was 29.3%, thus indicating good washing resistance and excellent flame-retardant durability.

Considering the concern over toxicity and environmental issues relevant to flame-retardant modification on textiles, abundant and green biomass materials, such as chitosan, an amino polysaccharide obtained from the shells of crustaceans, renewable and highly biocompatible, has attracted extensive attention of flame-retardant applications. It has been proved that CS can be used as a char-forming and blowing agent [11,12]. Kundu constructed an intumescent flame-retardant coating by layer-by-layer assembly technology based on chitosan and sodium alginate to impart polyamide 66 (PA 66) flame retardancy [13]. The PHRR of the PA 66 coated with 5 quadralayer deposition had a reduction of 24%, and the melt-dripping phenomenon was suppressed. Furthermore, Kundu fabricated a flame-retardant coating via UV grafting of phosphorylated chitosan (PCS) and sol-gel process of organo-silane on polyamide 66 [14]. The combination of PCS and organo-silane treatment showed the best effect in lowering PHRR, with 30% reduction.

In this work, GMA was firstly UV-grafted onto the surface of PAN fabric according to our previous work [9,10]. Next, the GMA grafted PAN fabric (PAN-g-GMA) reacted with CS (PAN-g-GMA-g-CS). Finally, PAN-g-GMA-g-CS was phosphorylated with phosphorus acid catalyzed by urea to obtain flame-retardant PAN fabric (FR-PAN). The aim of the work is to construct a green chitosan-based intumescent flame-retardant coating onto PAN fabric through UV-induced graft polymerization and chemical modification. It is worth noting that the intumescent coating can make full use of the char-forming and blowing ability of chitosan and the catalytic carbonization of phosphorus-containing groups. In other words, the coating can greatly improve the flame-retardant performance of PAN fabric. The structure, flame-retardant properties, and thermal stability of the treated fabrics were studied.

## 2. Results

### 2.1. Materials

Polyacrylonitrile (PAN) fiber containing 5 wt% vinyl acetate and 95 wt% acrylonitrile was supplied by Jilin Chemical Fiber Co., Jilin, China. Scoured plain-woven PAN fabric (150 g/m^2^) was woven by the School of Textiles Science and Engineering, Tianjin Polytechnic University, Tianjin, China. Glycidyl methacrylate (GMA) was purchased from Nanjing Xiezun Chemical Reagent Technologies Co., Ltd., Nanjing, China. Benzophenone (BP), chitosan (CS), phosphorus acid (85%), and urea were obtained from Tianjin Guangfu fine Chemical Research Institute, Tianjin, China. Methanol and acetone were purchased from Tianjin Fengchuan Chemical Reagent Technologies Co., Ltd., Tianjin, China. All the reagents were analytical grade and used without any further purification.

### 2.2. Preparation of GMA-Grafted PAN Fabric (PAN-g-GMA)

The preparation process is as follows: First, polyacrylonitrile fabric was washed with 10 wt% acetone aqueous solution for 30 min to remove the impurities adhering to the PAN fabric. Next, the fabric was rinsed with deionized water 3 times and dried to a constant weight. The dried fabric was cut into the size of 5 cm × 5 cm and dipped into a methanol solution which contains 2 wt% of BP and 35 wt% of GMA for 2 h. Then, the fabric was set under a UV lump with the wavelength of 365 nm and the distance of 15 cm for 20 min. Finally, the fabric was vigorously rinsed with distilled water to remove the excess BP, unreacted GMA, and its homopolymers. At last, the fabric was dried in an oven at 60 °C to a constant weight. The grafted percentage of GMA was calculated by the following equation:

*GP* (%) = [(*W*_2_ − *W*_1_)/*W*_1_] × 100% (1)

where *W*_1_ and *W*_2_ are the weight of the fabric before and after grafting, respectively.

Due to the fact that the higher grafting percentage of GMA will affect the feel of PAN fabric, in this study the PAN fabric with 30 wt% grafting percentage of GMA was selected for subsequent experiments.

### 2.3. Preparation of PAN-g-GMA-g-CS Sample

2 g of chitosan was dissolved in 98 g of acetic acid, then the PAN-g-GMA sample was immersed into the solution for 1 h at ambient temperature. Next, the solution was heated to 80 °C for 6 h to ensure the completed reaction between chitosan and epoxy groups of GMA. Finally, the CS grafted PAN-g-GMA sample (PAN-g-GMA-g-CS) was rinsed with distilled water for 3 times and dried in an oven at 60 °C for 12 h. The weight gain of PAN-g-GMA is about 5 wt%, i.e., the grafting percent of CS is 5 wt%.

### 2.4. Fabrication of FR-PAN Fabric

**Scheme 1:**
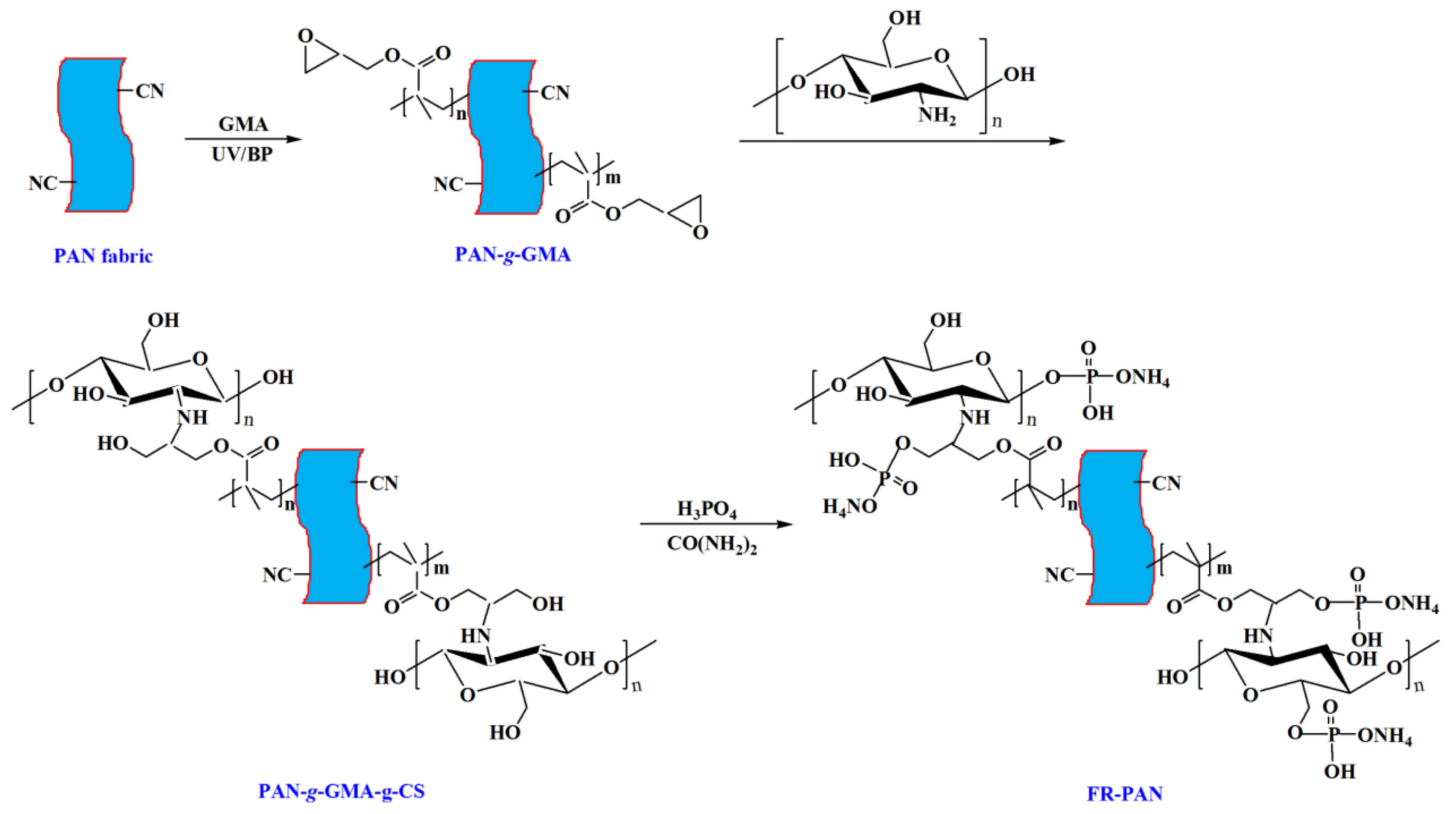
The schematic of preparing flame-retardant PAN fabric.

85 wt% of phosphorus acid was diluted with distilled water to a concentration of 60 wt%, and urea was added into the above solution at a bath ratio of 1:10, at the same time, the mixture was heated and stirred at 80 °C, and the PAN-g-GMA-g-CS sample was impregnated into the solution for 4 h with moderate stirring. Next, the flame-retardant PAN fabric (FR-PAN) was taken out, washed with deionized water and dried in a vacuum oven at 50 °C for 12 h. The preparation process of FR-PAN fabric is shown in Scheme 1.

### 2.5. Characterization

The chemical structure of different samples was characterized by Fourier transform infrared spectroscopy (FTIR) ranging from 500 to 4000 cm^−1^. The surface elemental compositions of the samples were analyzed by the X-ray photoelectron spectroscopy (XPS) with Al Kα radiation. The thermogravimetric analyses (TGA) of the fabrics were carried out under air atmosphere with a Q600 SDT thermogravimetric analyzer (Netzsch, Germany) from room temperature to 800 °C at a heating rate of 10 °C·min^−1^. The analysis of the gas species and the thermodynamic property of the samples was performed on a thermogravimetric analysis-infrared spectrometry (TG-FTIR) from room temperature to 800 °C with a heating rate of 10 °C·min^−1^. The heat capacity of the samples was recorded by a DSC 200 F3 (Netzsch, Germany) under nitrogen atmosphere from room temperature to 800 °C at a heating rate of 10 °C·min^−1^. The surface morphology of the samples before and after burning was examined by an S-4800 (Hitachi, Japan) scanning electron microscope (SEM). The samples were sputter-coated with gold layer before the testing. According to ISO 5660-1-2016, the burning performance of the FR-PAN fabric was investigated by a cone calorimeter (CC) (FTT, Tianjin Fire Research Institute) with 35 kW irradiation source. The limiting oxygen index (LOI) values of the samples were measured according to ASTM D6413-08 using an oxygen index apparatus.

## 3. Results

### 3.1. FTIR Analysis

**Figure 1:**
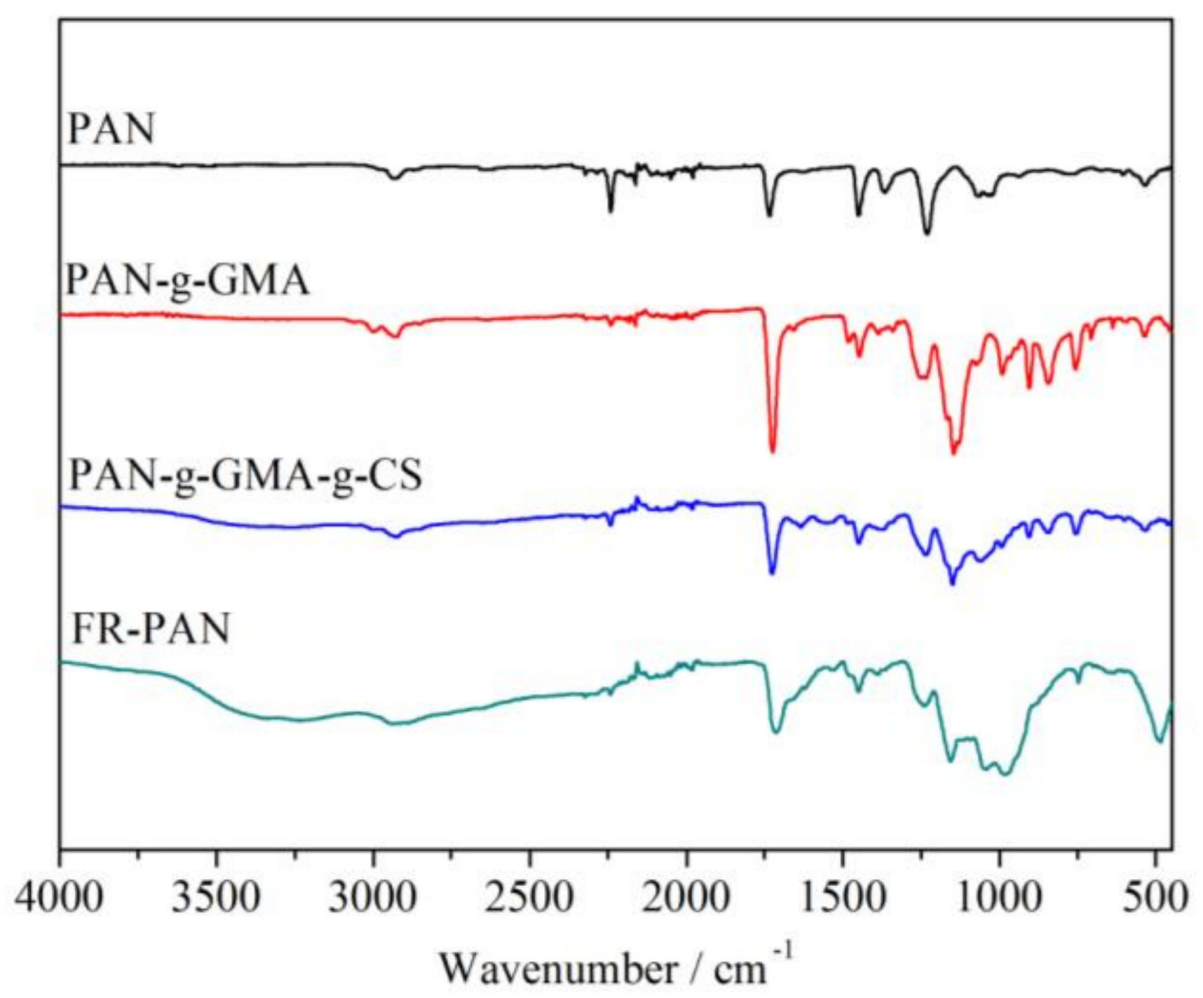
FTIR spectra of PAN and modified PAN fabrics.

FTIR spectra of the samples are shown in Figure 1. The grafting of GMA on the surface of PAN fabric was confirmed by the appeared epoxy group absorption peak at around 990 cm^−1^, 914–854 cm^−1^, and 764 cm^−1^ [15]. In addition, the adsorption peak at 1732 cm^−1^ of the PAN-g-GMA fabric is enhanced greatly owing to the grafted GMA. As for the PAN-g-GMA-g-CS sample, the appeared absorption peaks at around 1066 cm^−1^ and 3391 cm^−1^ attributing to the C-N and N-H, which indicate that CS was successfully grafted onto the PAN-g-GMA fabric. As for the spectra of FR-PAN, the bands at 1047 cm^−1^ and 974 cm^−1^ attributed to the characteristic peaks of P=O and P-O-C groups confirm the phosphorylation of PAN-g-GMA-g-CS sample [7,8,9,10]. Meanwhile, a wide peak appeared at around 3327 cm^−1^ ascribing to N-H group owing to the presence of NH^4+^ in the flame-retardant PAN fabric, as shown in Scheme 1.

### 3.2. XPS Analysis

**Figure 2:**
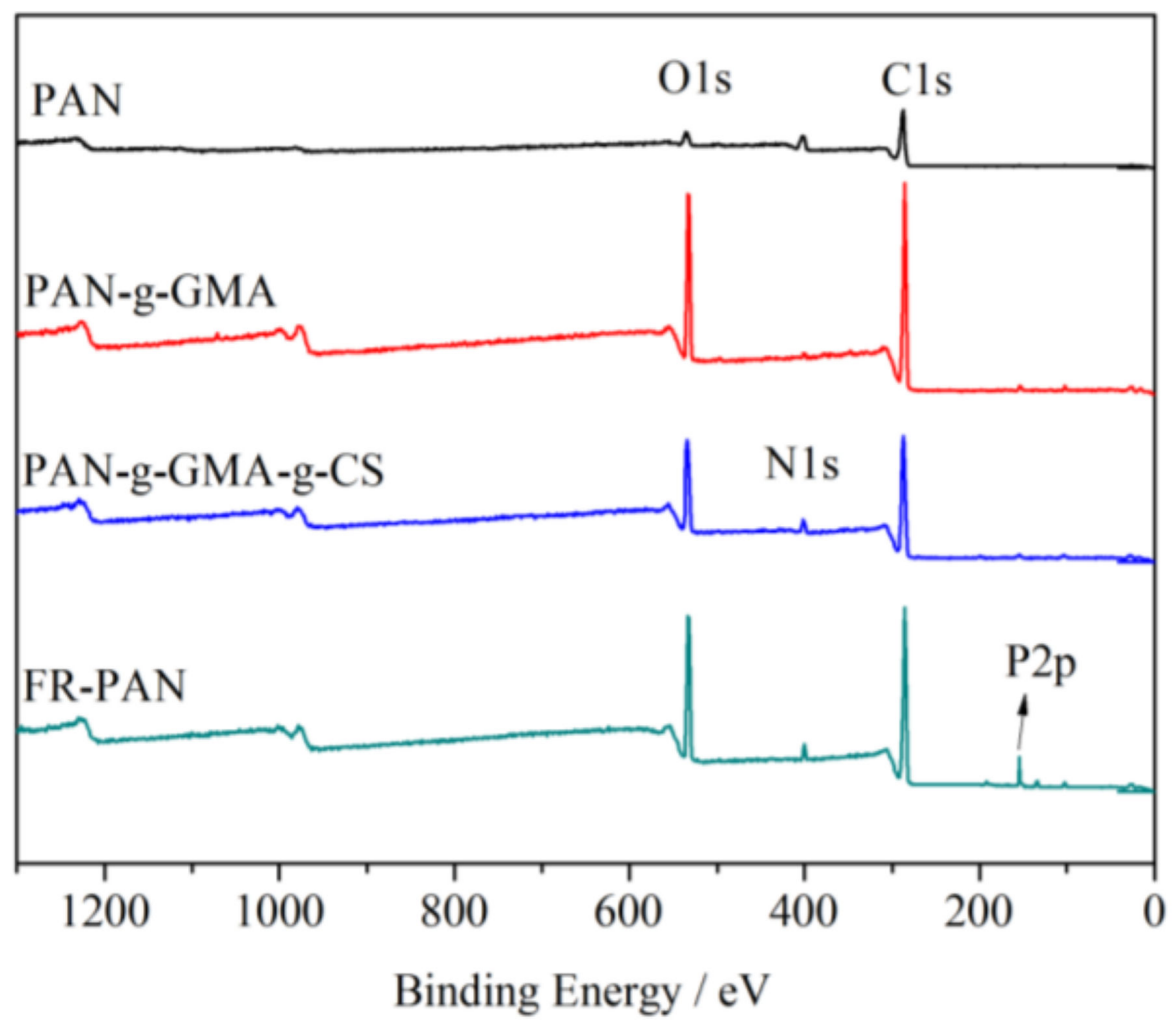
XPS spectra of PAN and modified PAN fabric.

The surface chemical compositions of the samples were examined by X-ray photoelectron spectroscopy, as shown in Figure 2. All of the samples showed three typical absorption peaks at 532, 405, and 286 eV, which respectively correspond to O1s, N1s, and C1s. As for the PAN-g-GMA fabric, the peaks of O1s and C1s are significantly increased compared with the blank sample, which is attributed to the grafted GMA. For the PAN-g-GMA-g-CS sample, the peak at 405 eV of N1s increases a little ascribing to the grafting of CS. For the FR-PAN, a new relative strong peak appeared at 134 eV owing to P2p [16], thus indicating the phosphorylation of PAN-g-GMA-g-CS.

### 3.3. TG-DTG Analysis

**Figure 3:**
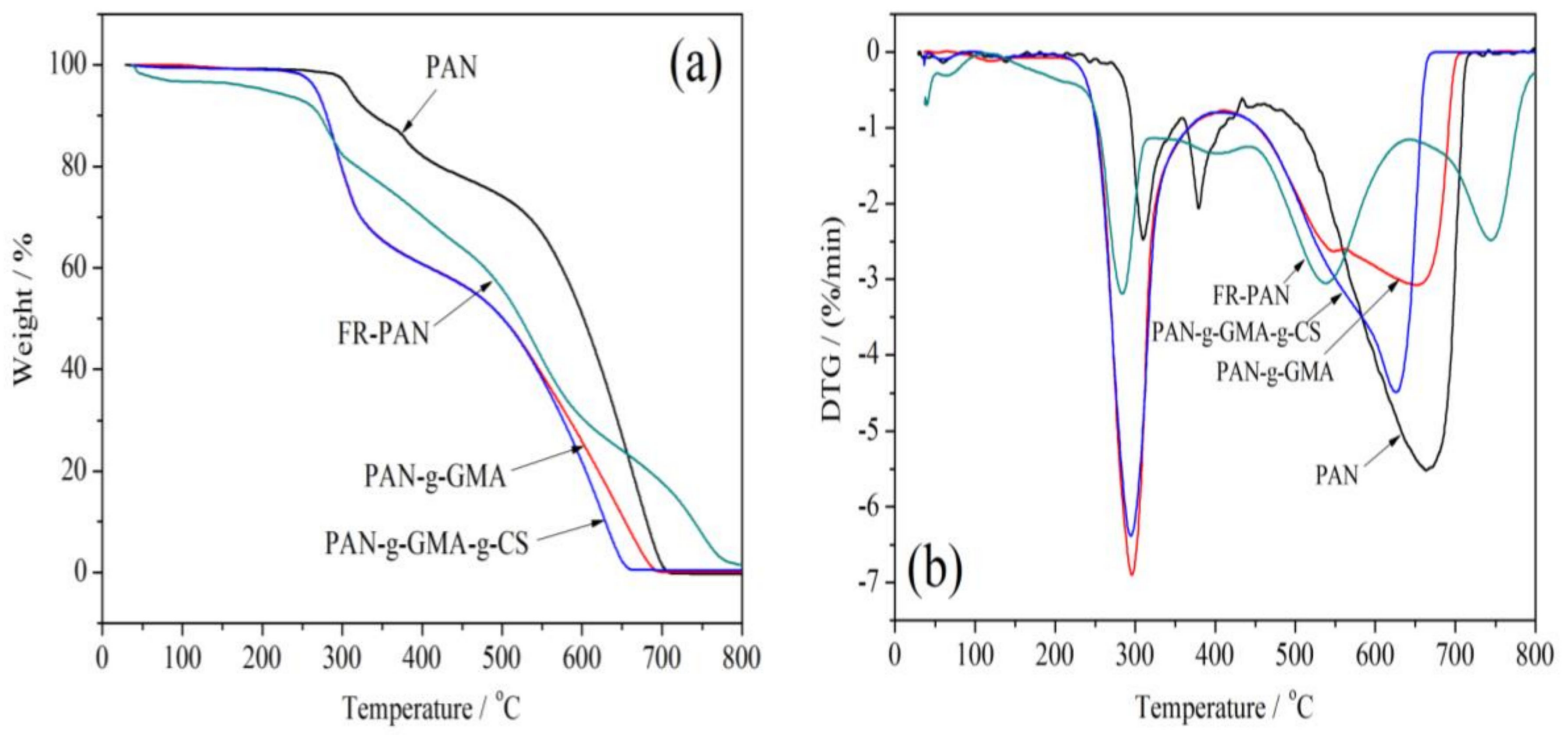
TG (**a**) and DTG (**b**) curves of PAN and modified PAN fabrics.

The thermogravimetric (TG) and the differential thermal gravimetric (DTG) curves of the samples are shown in Figure 3. For the virgin fabric, three obvious weight loss steps were observed, which correspond to the cyclization, decomposition–carbonization, and thermo-oxidation of the char [17,18]. The first weight loss occurs at around 282 °C with about 13 wt% mass loss, which results from the oligomerization of the adjacent cyano, the side groups decomposition of PAN, as a result, the gases such as ammonia and nitriles are released [19]. The second weight loss step begins from 360 °C to 430 °C due to the carbonation and decomposition of the cyclic structure. Among these reactions, a certain amount of gases, such as ammonia, hydrogen cyanide, and hydrogen are produced [20]. The third weight loss step occurs at above 480 °C, ascribing to the release of hydrogen and the further oxidation of the char produced in the first two stages.

For the PAN-g-GMA, the TG curve has two weight loss steps, the first weight loss step lies between 229 °C and 349 °C, and the peak temperature is about 297 °C. The initial decomposition temperature is lower than that of the origin fabric, which may be due to the earlier degradation of the grafted GMA chain. The second weight loss step is between 433 °C and 700 °C. The weight loss of this stage is due to the further degradation of GMA chain, the release of ammonia, hydrogen cyanide, hydrogen, and the oxidation of the char produced in the first stage.

The TG curve of the PAN-g-GMA-g-CS fabric is similar to that of the PAN-g-GMA sample because of the lower grafting percentage of CS (5 wt%). The difference between the two samples is that the second decomposition step of the PAN-g-GMA-g-CS sample is a little faster than that of the PAN-g-GMA sample. This may be due to the release of ammonia of CS units and the oxidation of the char.

Different from the other three samples, four decomposition stages of the FR-PAN fabric are observed. The initial decomposition begins from 120 °C, which is far lower than that of the other three samples, ascribing to the evaporation of the bounded water and the decomposition of the grafted GMA. The second weight loss step ascribes to the further decomposition of the grafted pendent groups and the dehydrogenation of cyanide cyclization. The third weight loss step may be due to the release of ammonia of CS units and the oxidation of the char. The last weight loss step attributes to the thermal oxidation decomposition of the char residue produced in the previous stages, as a result, some CO_2_ releases accordingly. On the one hand, the released non-flammable gases can dilute the oxygen concentration, on the other hand, they can isolate the contact between the matrix fabric and oxygen, as well as energy exchange, so as to achieve flame-retardant purposes.

### 3.4. DSC Analysis

**Figure 4:**
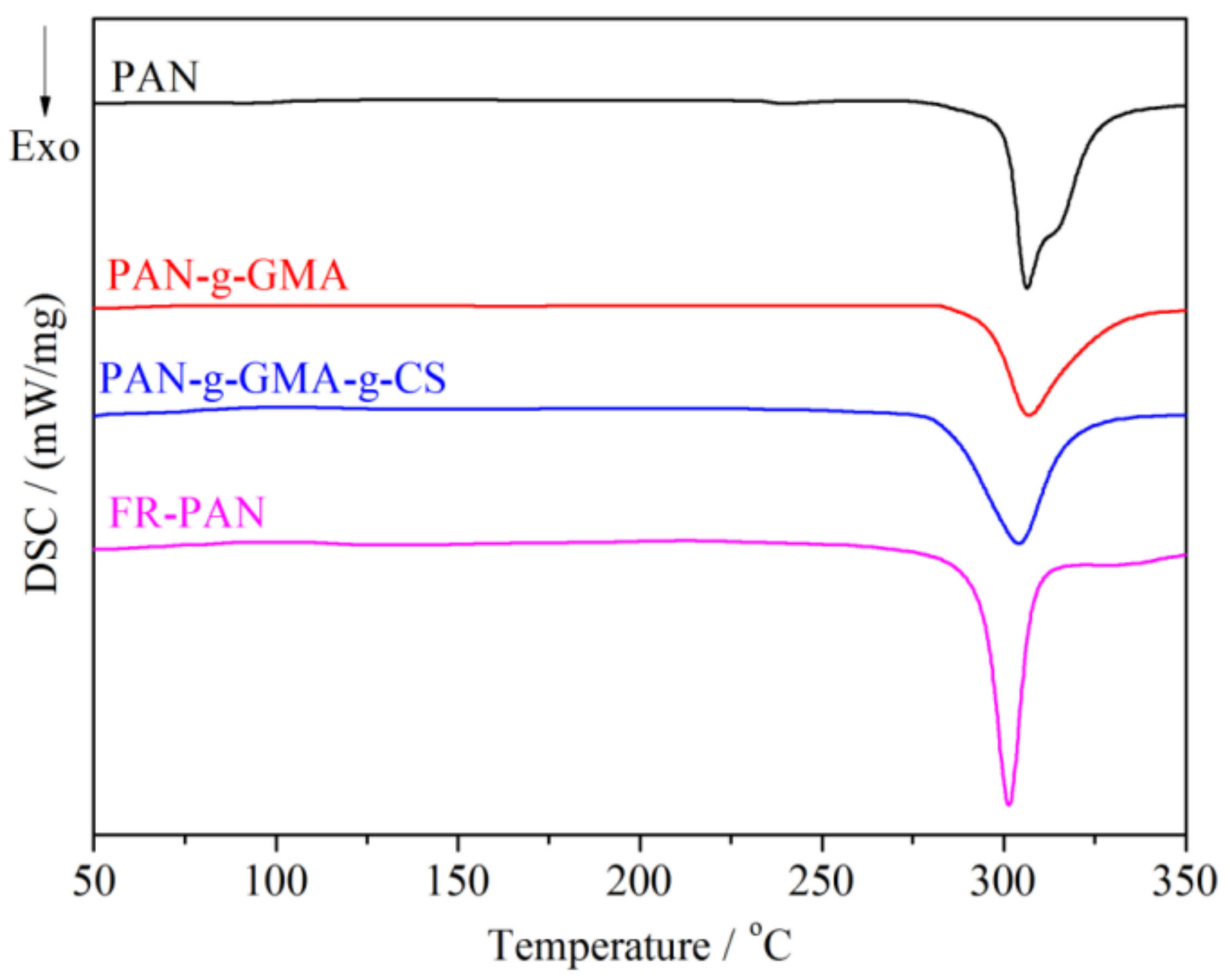
DSC curves of PAN and modified PAN fabrics.

The DSC plots of the samples were carried out in nitrogen atmosphere from ambient temperature to 350 °C so as to ensure no oxidation reaction occurred during the test progress, as shown in Figure 4. For the blank fabric, the initial exothermic peak starts from 275 °C, and the exothermic peak temperature locates at 306 °C, which is coincided with the TG analysis. This is due to the cyclization reactions initiated by a free radical mechanism [21]. For the PAN-g-GMA sample, the exothermic peak temperature is the same as the control sample, but the cyclization exothermic peak becomes weak. This may be due to the fact that the grafted GMA groups weaken the interaction of nitrile groups, thus the cyclization reaction of cyano groups was stunted. For the PAN-g-GMA-g-CS sample, the plot shape is similar to that of the PAN-g-GMA sample, and the exothermic peak lies at 304 °C. As for the FR-PAN sample, the exothermic peak begins from 262 °C, which is lower than those of the other three samples, and the exothermic peak situates at 301 °C. Moreover, the peak becomes stronger and sharper. This may be explained by the phosphorus-containing acids produced through the decomposition of the phosphate groups accelerating the cyclization reaction of the cyano groups [22,23].

### 3.5. TG-IR Analysis

**Figure 5:**
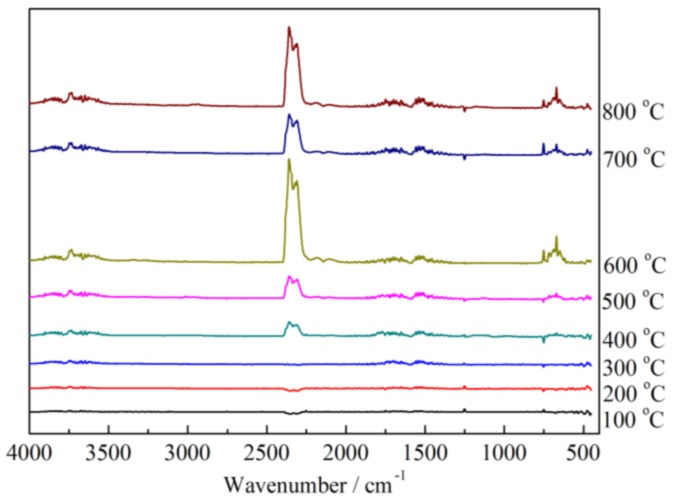
FTIR spectra of FR-PAN under different decomposition temperatures.

The gas species released from the thermal decomposition process under air atmosphere was characterized by using thermogravimetric analysis-infrared spectrometry (TG-IR). The FTIR spectra under different temperatures of the FR-PAN are shown in Figure 5. As shown in Figure 5, the major gases released from the thermal degradation of the FR-PAN sample are water or ammonia, alkanes, hydrogen cyanide, and carbon dioxide, corresponding to the peaks at 3750 cm^−1^, 2970 cm^−1^, 2248 cm^−1^, and 1760 cm^−1^. The peak at 670 cm^−1^ attributed to the bending vibration of -CH is not obvious at 100 °C and 200 °C. When the temperature rises to 300 °C, the peak becomes obvious, which is attributed to the early degradation of the grafted GMA and CS groups. Similarly, the peak at 750 cm^−1^ also attributes to the bending vibration of -CH. The peak at 1280 cm^−1^ is very obvious at 700 °C, this is mainly due to the release of the P=O or P-O free radical. The peak at 1760 cm^−1^ appears at different temperatures, attributed to the bending vibration of C=O resulting from the release of CO_2_.

### 3.6. Combustion Properties

**Figure 6:**
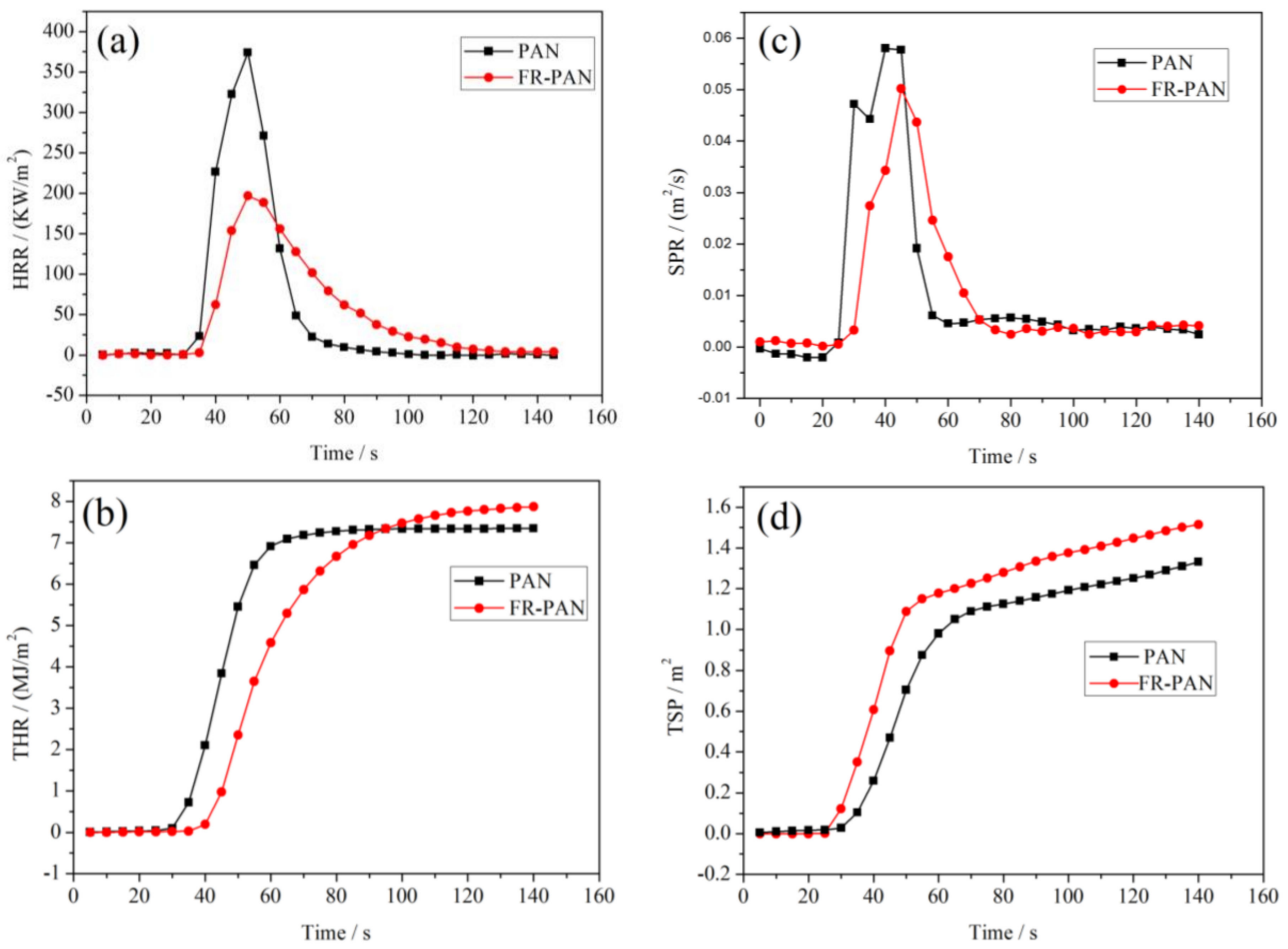
HRR (**a**), THR (**b**), SPR (**c**), and TSP (**d**) curves of PAN and FR-PAN fabrics.

Cone calorimetry (CC) is an effective instrument that can fully characterize the combustion properties of materials. Herein, we used it to research the combustion behavior of the blank PAN fabric and FR-PAN fabric, as shown in Figure 6 and Table 1. As seen from Figure 6a and Table 1, compared to the control sample, the peak of the heat release rate (PHRR) of the FR-PAN fabric decreases from 374.4 kW/m^2^ to 196.8 kW/m^2^ with 47.4% reduction. The total heat release (THR) of the FR-PAN fabric is 7.0 MJ/m^2^ showing a decrease compared to that of the blank sample. The peak of smoke production rate (PSPR) of the FR-PAN fabric (0.05 m^2^/s) is lower than that of the original fabric (0.06 m^2^/s). These indicates that the FR-PAN fabric has an excellent flame-resistant performance. However, the total smoke production (TSP) of the FR-PAN sample is 1.7 m^2^, which is higher than that of the blank sample (1.5 m^2^). This may be due to the incomplete combustion resulting from the good flame-retardant performance of the FR-PAN fabric, in other words, the treated fabric plays an effective flame-retardant role during combustion, thus the FR-PAN fabric produces much smoke. This can be verified by the higher char residue and the lower mass loss rate (aMLR). In detail, the average mass loss rate of the FR-PAN fabric is far lower than that of the virgin sample, and the char residue of the FR-PAN sample is much higher than that of the control fabric. In addition, the time to ignite (TTI) of the FR-PAN fabric increases to 34 s with 36% increase. Besides, the fire growth rate index (FIGRA) of the FR-PAN fabric is 5.94 with 28.6% reduction, thus indicating excellent fire safety. In summary, the results show that the flame-retardant properties of the FR-PAN fabric prepared in this work are excellent in preventing fabric burning. That is to say, the FR-PAN fabric prepared via UV-induced graft coating based on CS presented an outstanding flame resistance.

### 3.7. SEM Analysis

**Figure 7:**
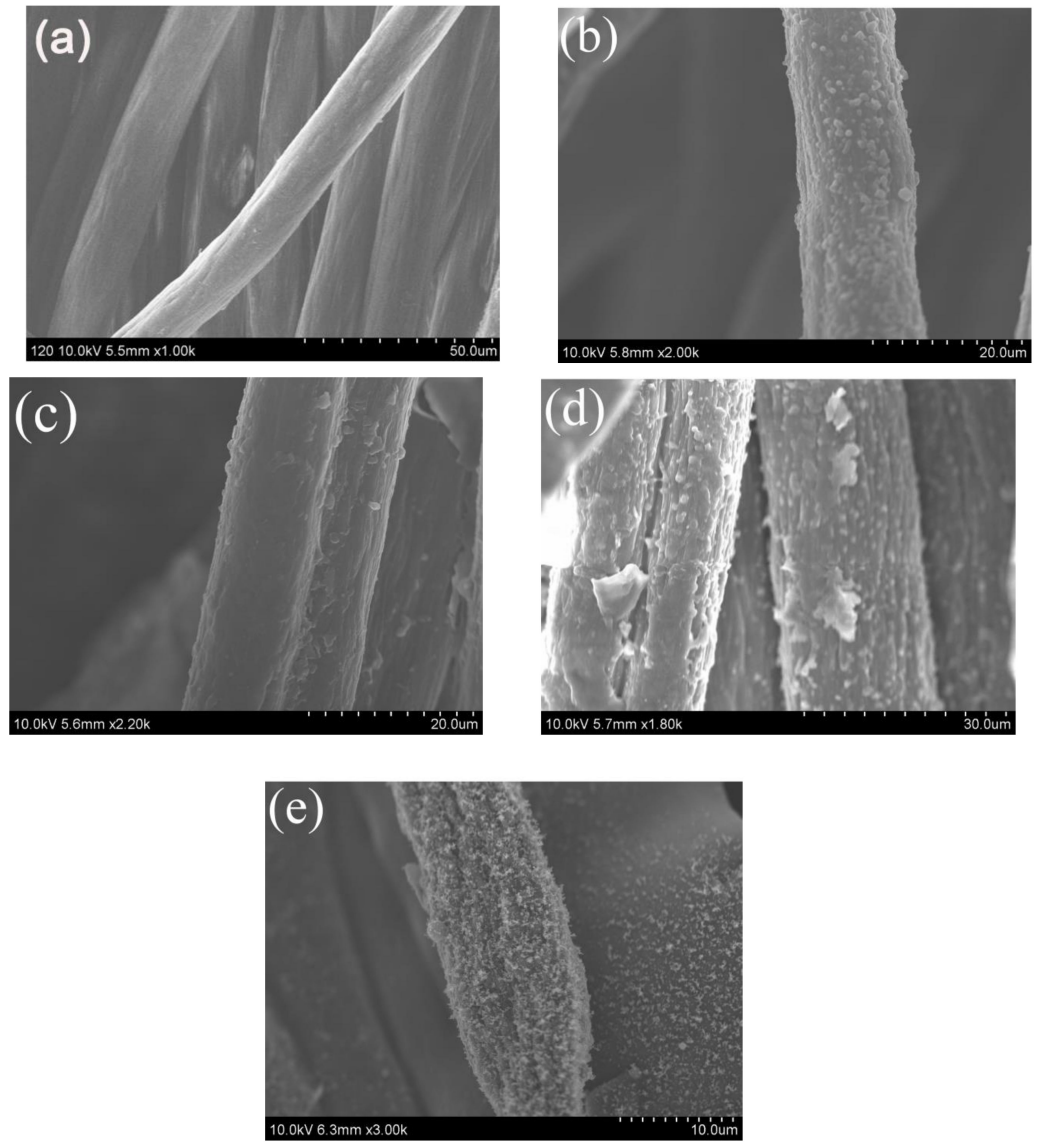
SEM photos of (**a**) PAN; (**b**) PAN-g-GMA; (**c**) PAN-g-GMA-g-CS; (**d**) FR-PAN; (**e**) char residue of FR-PAN.

Scanning electron microscope (SEM) was utilized to analyze the morphology of the different samples, as shown in Figure 7. The fibers of the blank fabric (Figure 7a) have a smooth and uniform surface with some grooves. Compared to the blank sample, the PAN-g-GMA fabric (Figure 7b) becomes rougher and some small particles adhere to the fabric owing to the grafted GMA chains. For PAN-g-GMA-g-CS (Figure 7c), the particles on the surface of the PAN fibers are disappeared, on the contrary, the surface becomes relative smooth and a thicker layer covers on the surface of the fibers. As for the FR-PAN fabric (Figure 7d), many particles appear on the surface of the fibers again, especially some large particles which stick out on the surface of the fibers due to the phosphorylation. For the char residue of the FR-PAN fabric (Figure 7e), many small dense protuberances appear on the surface of fibers. This may be due to the degradation and carbonization of the grafted chains during the combustion decomposition of the FR-PAN fabric, which in turn prevents further combustion decomposition of the fabric. It is pointed out that the char residue is compact and hard, which keeps the shape of the fabric, indicating that it has a good condensed phase flame-retardant effect. Meanwhile, the released nonflammable gases not only dilute the concentration of oxygen, but also isolate the fabric from oxygen and interrupt energy exchange, which greatly improves the flame-retardant properties of the FR-PAN fabric.

## 4. Conclusions

In this work, a chitosan-based flame-retardant coating was successfully performed on the surface of the PAN fabric through UV-grafting technology combined with chemical modification. The TG results indicate that the thermal stability of the FR-PAN fabric decreases compared with the control sample, however, the thermal degradation rate decreases accordingly. SEM results show that the coating is obvious, which plays an important role in insulating air and heat, preventing the release of the combustible gases, and avoiding further burning of the fabric. In addition, the cone calorimeter (CC) test results exhibit that the PHRR, THR, PSPR, and TSP of the FR-PAN fabric are lower than those of the original PAN fabric. Interestingly, the TTI and time to PHRR increase, especially the char residue increases greatly, thus indicating excellent flame-retardant performance.

## Figures and Tables

**Table 1:** Parameters of PAN and FR-PAN by cone calorimetry test.

Samples	TTI (s)	PHRR (kW/m^2^)	Time to PHRR(s)	THR (MJ/m^2^)	PSPR (m^2^/s)	TSP (m^2^)	aMLR (g/s)	Residue (wt%)	FIGRA (W/s)
PAN	25	374.4	45	8.6	0.06	1.5	0.02	38.0	8.32
FR-PAN	34	296.8	50	7.0	0.05	1.7	0.003	97.21	5.94
